# Trends of Utilization of Antiseizure Medications Among Pregnant Women in Manitoba, Canada: A 20-Year Population-Based Study

**DOI:** 10.3389/fphar.2022.871136

**Published:** 2022-04-20

**Authors:** Walid Shouman, Joseph A. Delaney, Kaarina Kowalec, Marcus Ng, Chelsea Ruth, Jamieson Falk, Christine Leong, Silvia Alessi-Severini, Alekhya Lavu, Payam Peymani, Sherif Eltonsy

**Affiliations:** ^1^ College of Pharmacy, Rady Faculty of Health Sciences, University of Manitoba, Winnipeg, MB, Canada; ^2^ Department of Epidemiology, University of Washington, Seattle, WA, United States; ^3^ Department of Medical Epidemiology and Biostatistics, Karolinska Institutet, Solna, Sweden; ^4^ College of Medicine, Rady Faculty of Health Sciences, University of Manitoba, Winnipeg, MB, Canada; ^5^ Department of Psychiatry, College of Medicine, Rady Faculty of Health Sciences, University of Manitoba, Winnipeg, MB, Canada; ^6^ Children’s Hospital Research Institute of Manitoba, Winnipeg, MB, Canada

**Keywords:** utilization, pregnancy, antiepileptic, cohort, epilepsy

## Abstract

**Background:** Evidence from developed countries demonstrates that the use of antiseizure medications (ASMs) has been increasing in the last decade. Pregnant women have a very challenging risk benefit trade-off in terms of ASM utilization, and it is crucial to know if increased utilization is seen among pregnant women.

**Objective:** To examine time-trends of utilization of ASM therapies among pregnant women in Manitoba, Canada.

**Methods:** We conducted a population-based cohort study using de-identified, linked administrative databases from Manitoba. Pregnancies between 1995 and 2018 were included. Four groups of pregnant people were created based on ASM exposure and epilepsy diagnosis.

**Results:** Of 273,492 pregnancies, 812 (3/1000) had epilepsy diagnosis and were exposed to ASMs, 963 (3.5/1000) had epilepsy diagnosis and were unexposed, and 2742 (10/1000) were exposed to ASMs and did not have epilepsy diagnosis. Overall, the number of pregnancies exposed to ASMs increased significantly from 0.56% in 1997 to 2.21% in 2018 (*p* < 0.0001). Subgroup analysis by epilepsy diagnosis showed no significant change in ASMs exposure among pregnant women with epilepsy [the proportion of women exposed to ASM from all pregnancies was 0.37% (in 1997) and 0.36% (in 2018), *p* = 0.24]. A drop in carbamazepine use was observed, while the number of lamotrigine prescriptions increased from 6.45% in 1997 to 52% by 2018. ASM use among pregnant women without epilepsy increased significantly from 0.19% in 1997 to 1.85% in 2018 (*p* < 0.0001). In the total cohort of pregnancies, 1439 (0.53%) were exposed during their entire pregnancy, and 1369 (0.5%) were exposed only in their first trimester. Clonazepam was the most used ASM during the study period (1953 users, 0.71%), followed by gabapentin (785 users, 0.29%) and carbamazepine (449 users, 0.16%).

**Conclusion:** No major shifts in the quantity of ASM use over the study period were observed among pregnant women with epilepsy. However, there was a significant increase in ASM use among pregnant women without epilepsy. The study results warrant further investigation into the implications of ASM use in pregnancy for indications other than epilepsy.

## Introduction

The estimated prevalence of epilepsy among pregnant women ranges between 0.3 and 0.7% (Hauser et al., 1996; Whelehan and Delanty, 2019). Both epilepsy and antiseizure medications (ASMs) are associated with potential adverse effects to a pregnant person and their developing fetus (Pennell, 2016; Whelehan and Delanty, 2019). Pharmacological management with ASMs during pregnancy should be maintained at the lowest possible dose allowing for optimum seizure control and minimal fetal exposure (Patel and Pennell, 2016; Pennell, 2016). Worldwide, several studies have reported an increase in the use of ASMs for epilepsy and other indications such as neuropathic pain, other neurologic and psychiatric disorders, and movement disorders (restless leg syndrome) during pregnancy (Vajda et al., 2010; Kulaga et al., 2011; Bobo et al., 2012; Wen et al., 2015; Yeh et al., 2017; Kinney et al., 2018; Hurault-Delarue et al., 2019; Margulis et al., 2019; Cohen et al., 2020). In a recent study, the utilization trends of ASMs during pregnancy from five Nordic countries, the United States, and Australia were assessed between 2006 and 2016 (Cohen et al., 2020). A significant increase in the use of ASMs, particularly new generation ASMs such as lamotrigine, and a decrease in old generation ASMs such carbamazepine during pregnancy was found in all countries throughout the study period (Cohen et al., 2020). In Canada, a study from the province of Québec by Kulaga et al. (2011) found that the majority of pregnant women with epilepsy (79.6%) received ASM monotherapy, 5.8% received polytherapy, and 14.6% had no ASM exposure. Evidence shows that the adverse outcomes are dependent on the type of ASM used, the dose of fetal exposure at conception, and the trimester of exposure (Hill et al., 2010; Tomson and Battino, 2012; Pennell, 2016). Therefore, choosing the most appropriate ASM for women with epilepsy (WWE), with the lowest teratogenic risk is crucial (Hill et al., 2010; Tomson and Battino, 2012; Pennell, 2016).

In the Canadian province of Manitoba, evidence of an increase in ASM use among the general population exists (Leong et al., 2016). A study showed that ASM use increased significantly, from 8.3/1,000 to 23/1,000 between 1998 and 2013 (Leong et al., 2016). The study showed a 210% increase in ASM users with no epilepsy, and 55-fold increase in the use of gabapentin among users without epilepsy (Leong et al., 2016). The study, however, did not report subgroup analysis for the trends of utilization of ASMs in special populations, such as pregnant women (Leong et al., 2016). In the current study, we aim to examine the trends of utilization of ASMs during pregnancy and identify any changes in prescription patterns of ASM among pregnant people with epilepsy in Manitoba, Canada, between 1995 and 2019.

## Materials and Methods

### Data Sources and Design

A retrospective population-based cohort study was conducted using de-identified data from the province of Manitoba, Canada. We constructed a cohort of all pregnant women in Manitoba from 1 January 1997 to 31 March 2019, using the administrative databases for the provincial healthcare system from the Manitoba Research Data Repository at the Manitoba Centre for Health Policy (MCHP), University of Manitoba. The database repository is a secure data-rich environment containing person-level health information on the entire population of Manitoba. All records in the repository are de-identified; however, records are linkable at the individual and family levels using a scrambled health number attached to each record. For the current study, we used the following linked databases: (1) The Manitoba Health Insurance Registry (date of birth, sex, comorbidities); (2) Drug Program Information Network (DPIN), which includes drug names, brand names, and dispensation dates and captures the dispensation of all prescription drugs by community pharmacies in Manitoba regardless of the insurance coverage type (1995/96–2018/19); (3) Hospital Discharge Abstracts, which include records of all patients' hospital admissions with summaries for demographic data (1992/93–2018/19), (4) Medical Services Database, which includes physician claims used to identify diagnosis codes using the International Classification of Diseases (ICD-9 and ICD-10) (1992/93–2018/19), (5) The Hospital Newborn to Mother Link, which serves to match the baby's birth hospital record with the mother’s obstetrical delivery record, and (6) census data for income quintiles (IQ). All data sets were linked together using a scrambled personal health identification number that is unique for each mother (1995/96–2018/19). We conducted sensitivity analysis using diagnosis codes in 2, 5, and 10 years prior to pregnancy case. The 5 years' definition was used to minimize false-negative cases of epilepsy.

### Study Population

We identified all pregnancies for women living in Manitoba between 1995 and 2018. A woman was considered to have epilepsy if she had ≥1 medical claims or ≥1 hospitalization for epilepsy during the 5 years prior to delivery (ICD-9: 345 or ICD-10: G40/G41) (Fisher et al., 2014; Tu et al., 2014; Leong et al., 2016). Four groups of pregnant women were created: (1) exposed pregnant women with epilepsy, (2) exposed pregnant women without epilepsy, (3) unexposed pregnant women with epilepsy, and (4) unexposed pregnant women without epilepsy. Women who did not have five-year coverage or whose children were born before 1 April 1997 were excluded due to <5 years of follow-up. The area of residence was defined as urban for women living in Winnipeg or Brandon or as rural for women living in all other areas of the province. Income quintiles were used to determine the socioeconomic status. Income quintile measures neighborhood socioeconomic status and divides the population into five income groups from the lowest to the highest income (approximately 20% of the population in each group) (Martens et al., 2015).

### Exposure Definition

ASM utilization was identified using the Anatomical Therapeutic Chemical (ATC) codes. The exposure windows were first trimester (1^st^ day of gestation–14^th^ week), second trimester (15^th^ week–25^th^ week), third trimester (26^th^ week–end of pregnancy), and anytime during pregnancy (1^st^ day of gestation–end of pregnancy). Exposure to a prescribed ASM was defined as having ≥1 prescription filled during the exposure window of interest, or a prescription filled before the beginning of the exposure window but with a duration overlapping the exposure window. ASMs examined were identified using ATC codes within the prescription drug data, specifically all drugs coded as N03A for anti-epilepsy medication (Supplementary Table S1).

### Statistical Analysis

The characteristics and comorbidities of women were evaluated using descriptive statistics. Patient comorbidities considered (including mood disorders, diabetes, and hypertension) are defined in Supplementary Table S2. The frequency and pattern of ASM use during the whole pregnancy and each trimester was estimated. The annual trend of use of ASMs was evaluated for the total study population and for women with epilepsy and women without epilepsy. Linear regression was used to model the annual trends of utilization of ASMs and specific ASMs in each group of pregnant women. Models were built using data from 1997 to 2018 as some medications were only available as of 1997. A *p*-value ≤0.05 was considered statistically significant. Statistical analyses were performed using SAS software, version 9.4 (SAS Institute Inc., Cary, NC).

## Results

**FIGURE 1:**
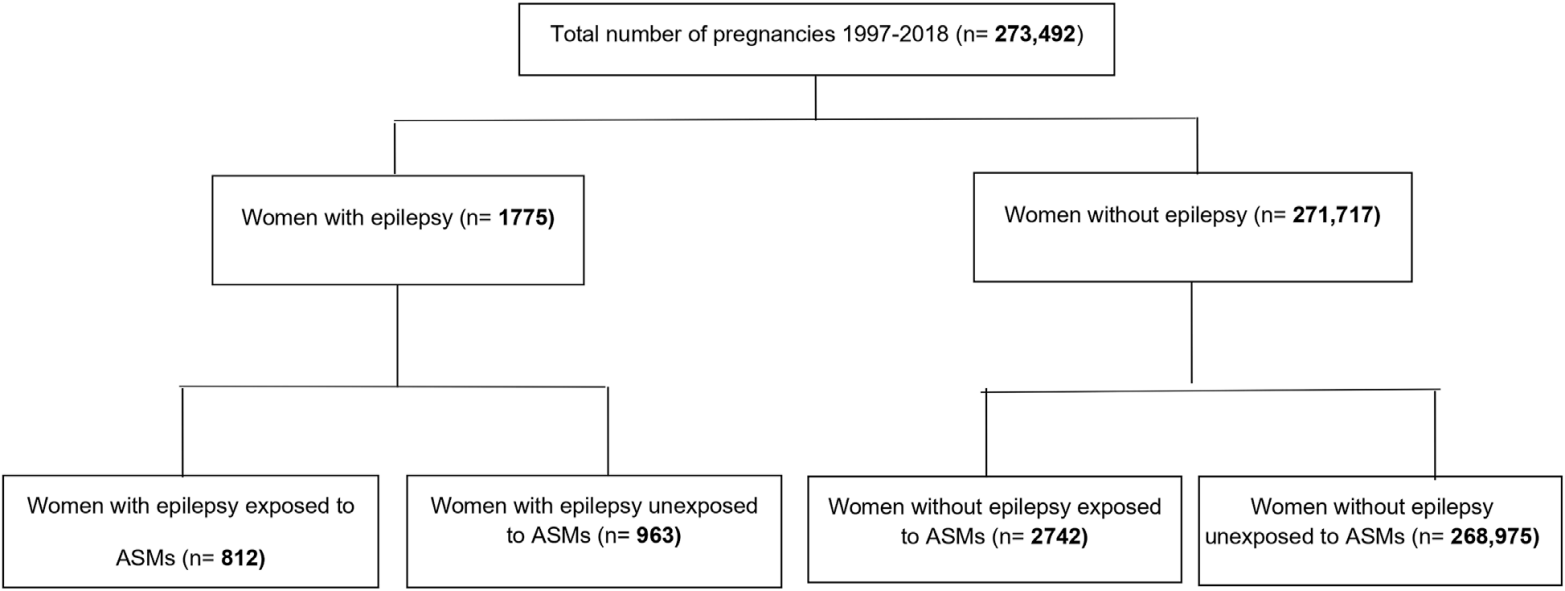
Study flowchart.

We identified 273,492 pregnancies, with a mean age of 28 years. Of these pregnancies, 0.3% (*n* = 812) were in women with epilepsy exposed to ASMs, 0.35% (n = 963) were pregnancies of women with epilepsy unexposed to ASM, and 1% (*n* = 2742) were women without epilepsy but exposed to ASMs (Figure 1). Among women with epilepsy, 31.3% (*n* = 254) of the exposed pregnancies and 31.3% (*n* = 301) of the unexposed pregnancies were in the lowest income quintile. Whereas, in women without epilepsy, 43.5% (*n* = 1193) of exposed pregnant women were in the lowest income quintile compared to 26.3% (*n* = 70812) in unexposed pregnant women (Table 1). Exposed pregnant women without epilepsy had higher rates of comorbidities compared to other groups. Among the exposed pregnant women without epilepsy, 65.21% were diagnosed with anxiety and 20.31% were diagnosed with pain when compared to 10.22 and 5.55%, respectively, in unexposed pregnant women without epilepsy (Table 1).

**TABLE 1:** Characteristics of the study population by group.

		Exposed		Unexposed	
		Pregnant women with epilepsy	Pregnant women without epileps*y*	Pregnant women with epilepsy	Pregnant women without epilepsy
Total, N (%)		812 (0.3%)	2,742 (1%)	963 (0.4%)	268,975 (98.4%)
Mean age (SD)		27.9 (±5.5)	29.2 (±5.6)	26.6 (±6)	28.1 (±5.8)
SES quartiles	1 (Lowest)	254 (31.3%)	1193 (43.5%)	301 (31.3%)	70812 (26.3%)
	2	214 (26.4%)	561 (20.5%)	203 (21.1%)	56928 (21.2%)
	3	153 (18.8%)	434 (15.8%)	196 (20.4%)	49153 (18.3%)
	4	110 (13.6%)	286 (10.4%)	166 (17.2%)	49483 (18.4%)
	5 (Highest)	75 (9.2%)	258 (9.4%)	95 (9.9%)	41820 (15.6%)
Area of residence	Rural	357 (44.0%)	1029 (37.5%)	387 (40.2%)	125655 (46.7%)
	Urban	449 (55.3%)	1703 (62.1%)	574 (59.6%)	142541 (53.0%)
Hypertension, N (%)		27 (3.3%)	227 (8.3%)	34 (3.5%)	4212 (1.5%)
Diabetes, N (%)		26 (3.2%)	212 (7.7%)	34 (3.5%)	7684 (2.9%)
Mood and anxiety disorders, N (%)		189 (23.3%)	1788 (65.2%)	208 (21.6%)	27481 (10.2%)
Schizophrenia, N (%)		10 (1.2%)	90 (3.3%)	suppressed	583 (0.2%)
Personality disorder, N (%)		34 (4.2%)	270 (9.9%)	38 (4.0%)	2638 (1.0%)
Pain, N (%)		86 (10.6%)	557 (20.3%)	122 (12.7%)	14932 (5.6%)
Birth status	Stillborn, N (%)	Suppressed	28 (1.0%)	8 (0.8%)	8 (0.8%)
	Singleton, N (%)	788 (97.0%)	2666 (97.2%)	945 (98.1%)	262111 (97.5%)
	Multiple births, N (%)	24 (3.0%)	76 (2.8%)	18 (1.9%)	6864 (2.6%)

**FIGURE 2:**
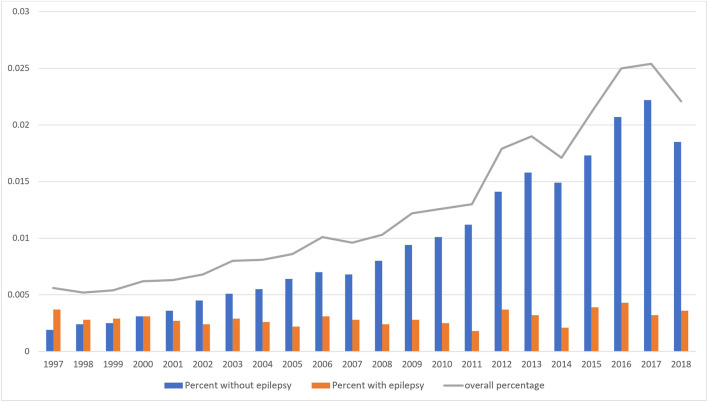
Annual Trend of utilization of ASMs among all pregnant women with and without epilepsy.

Linear regression analyses showed the number of pregnancies exposed to ASMs increased significantly from 0.56% in 1997 to 2.21% in 2018 (*p* < 0.0001) (Figure 2). There was no significant change in the percentage of pregnant women with epilepsy exposed to ASMs from 0.37% in 1997 to 0.36% in 2018 (*p* = 0.2354), while the percentage of ASM-exposures among pregnant women without epilepsy increased significantly (0.19% in 1997 to 1.85% in 2018, *p* < 0.0001) (Figure 2).

### Trimester Analysis

Trimester analysis showed 0.53% (*n* = 1439) of women were exposed throughout their pregnancy, 0.5% (*n* = 1369) were exposed only in the first trimester, 0.02% (*n* = 63) were exposed only during the second trimester, and 0.07% (*n* = 184) were exposed only during the third trimester. Among women with epilepsy, 33.58% were exposed throughout the pregnancy. Detailed analysis of exposures by trimester is presented in Supplementary Figures S1–S3.

**FIGURE 3:**
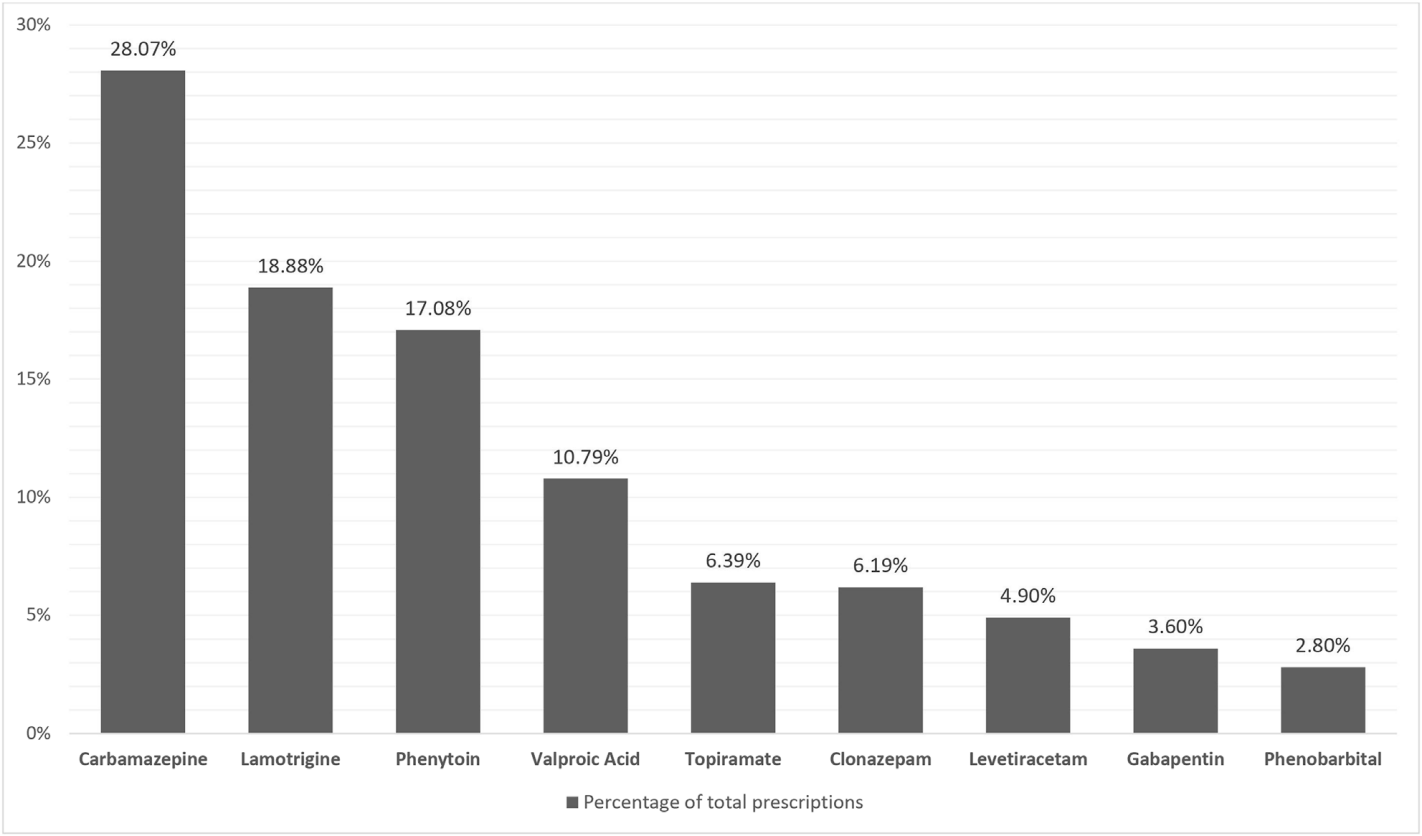
Most used ASMs among pregnant women with epilepsy.

The most used ASM among pregnant women with and without epilepsy, throughout the study period was clonazepam (44.44% of all exposed pregnancies) followed by gabapentin (17.85%) and carbamazepine (10.22%) (Table 2). Whereas, among pregnant women with epilepsy, carbamazepine (33.86%), lamotrigine (22.77%), phenytoin (17.08%), and valproic acid (13%) were the most used (Figure 3).

**TABLE 2:** Percentage of exposed pregnancies to each ASM by group.

	All exposed pregnant women (%)	Exposed pregnant women with epilepsy (%)	Exposed pregnant women without epilepsy
Clonazepam	45.88	6.19	59.67%
Gabapentin	18.38	3.6	23.52%
Carbamazepine	9.33	28.07	2.81%
Lamotrigine	7.86	18.88	4.03%
Levetiracetam	1.31	4.9	Suppressed
Valproic acid	5.46	10.79	3.61%
Phenytoin	5.44	17.08	1.39%
Topiramate	4.41	6.39	3.72%

Values ≤5 were suppressed.

**FIGURE 4:**
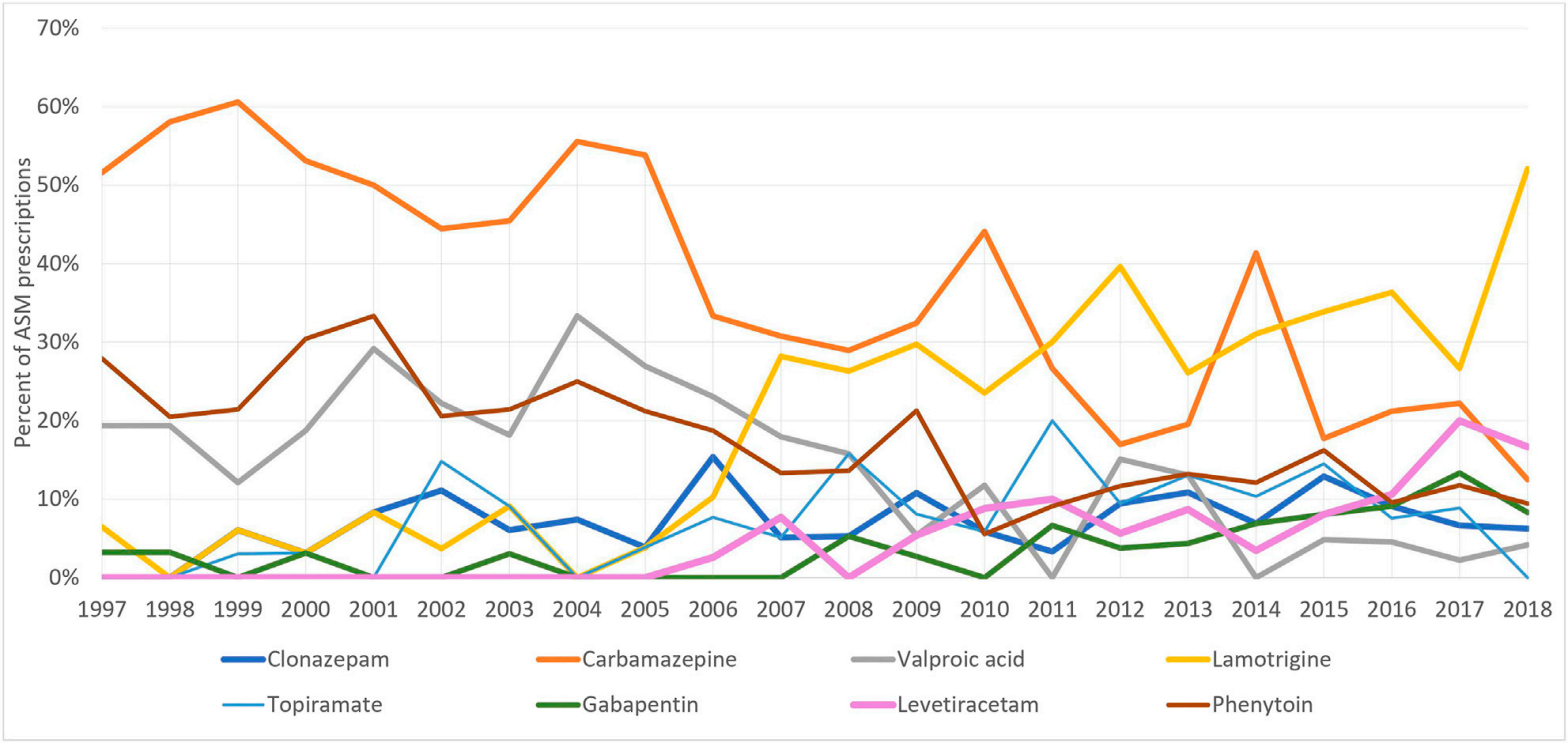
Trends of top ASM prescriptions among pregnant women with epilepsy.

**FIGURE 5:**
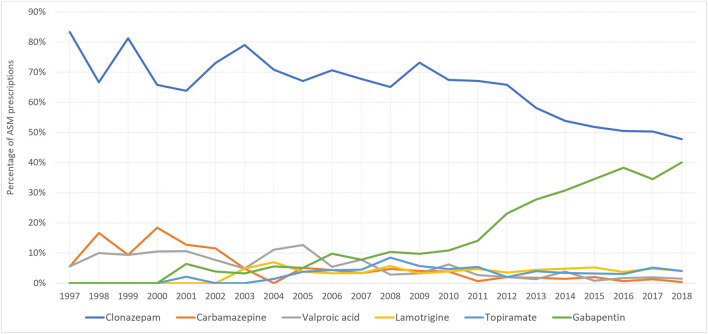
Trends of top ASM prescriptions among pregnant women without epilepsy.

At the start of the study period, carbamazepine was the most prescribed ASM for pregnant women with epilepsy (51%), however, this decreased to 12.5% in 2018, whereas the number of lamotrigine prescriptions increased from 6.45% (1997) to 52% (2018) (Figure 4). On the other hand, among women without epilepsy, clonazepam remained the most used ASM throughout the study period. However, its utilization decreased from 88.2% in 1997 to 47.97% in 2018. Gabapentin first appeared among women without epilepsy in 2001 and its utilization increased to reach 40.2% of prescriptions in 2018 (Figure 5).

## Discussion

In this population-based cohort study, we observed a significant increase in the utilization of ASMs among pregnant women in the Canadian province of Manitoba between 1997 and 2018. This increase was attributed mainly to the increased use of clonazepam and gabapentin among pregnant women without epilepsy. In general, there was no major shift in the utilization of ASMs among pregnant women with epilepsy over the study. By contrast, a significant increase in the utilization of ASMs among pregnant women without epilepsy was observed. Our study showed an increase in lamotrigine prescriptions among pregnant women with epilepsy and a decrease in valproic acid and carbamazepine use. Similar results were reported in the United Kingdom and Ireland, with an increase in lamotrigine and levetiracetam use and a decrease in valproic acid and carbamazepine between 1996 and 2016 (Kinney et al., 2018). Lamotrigine prescriptions increased from 15% of the total ASM prescriptions in the United Kingdom and Ireland in 2000 to 31% in 2016, while at the same time, valproic acid prescriptions decreased from 22% in 2000 to less than 5% in 2016 (Kinney et al., 2018). ASMs are frequently used for indications other than seizure control (LiverTox, 2012; Dokkedal-Silva et al., 2019). For example, valproic acid has been indicated for bipolar disorder and schizophrenia (LiverTox, 2012; Dokkedal-Silva et al., 2019). Lamotrigine has been indicated in bipolar depression in adults (Goldenberg, 2010). Gabapentin is primarily used to treat neuropathic pain, namely, trigeminal neuralgia, HIV-associated neuralgia, diabetic neuropathy, and neoplasia (Magnus, 1999; Goldenberg, 2010). It is also used in the treatment of psychiatric disorders, most notably bipolar disorder, and in movement disorders such as restless leg syndrome (Magnus, 1999; Goldenberg, 2010). Most exposed pregnant women with epilepsy (33.6%) were exposed throughout their pregnancy period, and while the main reasons are unknown, this could be a reflection to optimal management of seizures by practitioners. Among the pregnant women with epilepsy, >54% were unexposed to any ASM, this could be attributed to the presence of mild/non–medication-controlled epilepsy, or a potential misclassification of epilepsy definition used in our study (for example, isolated seizures not related to epilepsy).

### Strengths and Limitations

The databases used in this study are a major strength in terms of size and coverage, and the validity and reliability of the MCHP Repository for epidemiological studies has been previously reported (Leong et al., 2016; Azimaee et al., 2018). The MCHP repository includes medical records for all Manitoba residents recorded in the process of routine care. Our study captured the prescription practices of prescribers in Manitoba during the past 20 years. Our study, however, has limitations. First, exposure was derived from dispensing records and not actual intake (Azimaee et al., 2018). Second, we did not have data on the severity of epilepsy cases. Finally, since many prescriptions started prior to pregnancy, a proportion of women may have stopped taking their medications as soon as they become pregnant, without a database record, thus overestimating some ASMs exposures.

## Conclusion

Over the study period, no major shifts in the overall use of ASMs were observed among pregnant women with epilepsy. The reduction in carbamazepine and valproic acid use coupled with the increase in lamotrigine and levetiracetam use reflects Manitoba prescribers' adherence to updated guidelines (CCSO, 2015). Consistent with previous reports among the general population of Manitoba, gabapentin is increasingly used among pregnant women, mostly for non-epilepsy indications. Future studies on the utilization and safety outcomes of gabapentin and other new-generation ASMs in pregnancy, as well as studies focusing on pre-pregnancy counseling and management are warranted to inform prescribers and policymakers.

## Data Availability

The data sets presented in this article are not readily available because data used in this study are from the Manitoba Population Research Data Repository housed at the Manitoba Centre for Health Policy, University of Manitoba, and were derived from data provided by Manitoba Health. Requests to access the data sets should be directed to Manitoba Centre for Health Policy, https://umanitoba.ca/manitoba-centre-for-health-policy/.
