# Supplementary material for: Trends of Utilization of Antiseizure Medications Among Pregnant Women in Manitoba, Canada: A 20-Year Population-Based Study
Source: Front Pharmacol. 2022 Apr 20;13:871136. doi: 10.3389/fphar.2022.871136 (PMC9065250; doi:10.3389/fphar.2022.871136)
Supplement: Supplementary file 1 [file DataSheet1.docx]

**Supplementary**

**Supplementary Table 1. ASMs with N03A ATC Code**

| **ATC Code** | **Medications** |
| --- | --- |
| N03A | Phenobarbital |
|  | Primidone |
|  | Phenytoin |
|  | Ethosuximide |
|  | Mesuximide |
|  | Clonazepam |
|  | Carbamazepine |
|  | Oxcarbazepine |
|  | Valproic acid |
|  | Vigabatrin |
|  | Lamotrigine |
|  | Topiramate |
|  | Gabapentin |
|  | Levetiracetam |
|  | pregabalin |
|  | Lacosamide |

**Supplementary Table 2. Inclusion/Exclusion Criteria, Exposures, Outcomes and Definitions**^1–3^

| ***The medical conditions and comorbidities of the four groups were identified using ICD 9 codes from physician claims and hospitalizations and medication data.*** | |
| --- | --- |
| Epilepsy | A woman will be considered to have epilepsy if she has one (or more) hospitalization for epilepsy or physician billing visit (ICD-9=345 and ICD-10-CA*=G40/G41) during the 5 years prior to delivery. |
| Hypertension | - At least one hospital diagnosis for hypertension: (ICD-9-CM: 401-405 OR ICD-10-CA: I10-I13, I15); in year before conception OR at least two ambulatory visit diagnoses: (ICD-9-CM codes: 401-405) in the year before conception, OR at least two dispensations for hypertension medication in the year before conception. |
| Diabetes | Diabetes was defined as individuals with one of the following in 3-year period: one or more hospitalizations with a diagnosis of diabetes (ICD-9-CM code 250; ICD-10-CA codes E10-E14), or two or more physician visits with a diagnosis of diabetes (ICD-9-CM codes as above), or one or more prescriptions for medications to treat diabetes (ATC code A10). |
| Mood and anxiety disorders | A woman was considered to have prenatal mood and/or anxiety disorder (e.g. anxiety and/or depression) if in the 1 year prior to conception (or hospital discharge in case of a stillbirth) she had: one or more hospitalizations with a diagnosis for depressive disorder, affective psychoses, neurotic depression,  or adjustment reaction (ICD-9-CM codes 296.2-296.8, 300.4, 309, 311; ICD-10-CA codes F31, F32, F33, F34.1, F38.0, F38.1, F41.2, F43.1, F43.2, F43.8,F53.0, F93.0)  OR one or more physician visits with a diagnosis for depressive disorder, affective psychoses,  or adjustment reaction (ICD-9-CM codes 296, 309 or 311) OR one or more hospitalizations with a diagnosis for anxiety disorders (ICD-9-CM code 300; ICD-10-CA codes F32.0, F34.1, F40, F41, F42, F44, F45.0, F451, F452, F48, F68.0, F99)  or one or more hospitalizations with a diagnosis for anxiety states, phobic disorders, or obsessive- compulsive disorders (ICD-9-CM codes 300.0, 300.2, 300.3; ICD-10-CA codes F40, F41.0, F41.1, F41.3, F41.8, F41.9, F42)  OR two or more physician visits with a diagnosis for anxiety disorders (ICD-9-CM code 300). |
| Personality disorders | -One or more hospitalization with a diagnosis for personality disorders in one year before birth  ICD-9-CM code: 301; OR ICD-10-CA codes: F21, F34.0, F60, F61, F62, F68.1, F68.8 or F69; OR at least one physician visit with a diagnosis for personality disorders using ICD-9-CM code 301. |
| Pain | Pain disorders were defined as: diagnosis of pain (ICD-9 code 338; ICD-10 R52), or migraine and headaches (ICD-9 346 784; ICD-10 G43 R51) during the 1 year before conception. |
| Schizophrenia | During one year prior to conception:  Schizophrenia was defined as any women with one or more hospitalization with a diagnosis for schizophrenia (ICD-9-CM code: 295 (schizophrenic disorders), or ICD-10-CA codes: F20 (schizophrenia), F21 (schizotypal disorder), F23.2 (acute schizophrenia-like psychotic disorder), F25 (schizoaffective disorders), or one or more physician visits with a diagnosis for schizophrenia using ICD-9-CM code 295 in the 1 year prior to conception. |

* Canadian V of ICD10

1. Fransoo R, Mahar A, The Need To Know Team, Anderson A, Prior H, Koseva I, McCulloch S, Jarmasz J, B. S. *Fransoo R, Mahar A, The Need To Know Team, Anderson A, Prior H, Koseva I, McCulloch S, Jarmasz J, Burchill S.* ipeg, MB. Manitoba Centre for Health Policy.Autumn 2019.

2. Singal, D. *et al.* In Utero Antidepressants and Neurodevelopmental Outcomes in Kindergarteners. *Pediatrics* **145**, (2020).

3. Gaffney, A. *et al.* The Incidence of Diabetic Ketoacidosis During ‘Emerging Adulthood’ in the USA and Canada: a Population-Based Study. *J. Gen. Intern. Med.* **34**, 1244–1250 (2019).

Supplementary Figure 1. Percentage of pregnant women exposed to ASMs by trimester

Supplementary Figure 2. Percentage of pregnant women with epilepsy exposed to ASMs by trimester

Supplementary Figure 3. Percentage of pregnant women without epilepsy exposed to ASMs by trimester
